# Insulin use, prescription patterns, regimens and costs.-a narrative from a developing country

**DOI:** 10.1186/1758-5996-4-50

**Published:** 2012-12-02

**Authors:** Anthonia O Ogbera, Sonny F Kuku

**Affiliations:** 1 Department of Medicine, Lagos State University Teaching Hospital, Lagos State, Nigeria; 2 Eko Hospital Ikeja, Lagos State, Nigeria

## Abstract

**Background:**

Achieving good glycemic control is of paramount importance in the reduction of diabetes mellitus (DM) associated morbidity and mortality. Insulin plays a key role in the management of DM but unfortunately whilst some healthcare providers present insulin as a treatment of last resort , patients on insulin often have insulin related issues such as needle phobias, fear of hypoglycaemia, weight gain and in developing countries, costs. This Report aims at assessing insulin prescription pattern, insulin costs and issues associated with adherence.

**Methods:**

This was a Cross-sectional observation Study whereby 160 patients with DM who were on insulin solely or in combination with oral hypoglycaemic agents were recruited over a 6 month period. Information obtained from the Study subjects pertained to their histories of DM, types of insulin, insulin costs, adherence issues and insulin delivery devices. Long and short term glycaemic control were determined and evaluated for possible relation to insulin adherence. Test statistics used were chi square, t test and binary regression.

**Results:**

Insulin adherence was noted in 123-77% of the Study subjects and this was comparable between persons with type 1 DM and those with type 2 DM. The mean glycosylated haemoglobin values were significantly higher in those who admitted to non insulin adherence compared to those who adhered to their insulin regimen (9.7% (2.3) Vs 8.6% (2.1), p = 0.01). Reasons proffered by Respondents for non insulin adherence included high costs-15(41%), inconvenience −15 (41%) and needle pain-79)18%. A greater proportion of persons who self injected insulin adhered to insulin prescriptions compared to those who did not self inject and thus had better glycaemic control. Shorter duration of DM and older age were found to be predictors of adherence to insulin prescription.

The monthly mean costs of insulin for those who earned an income was 5212.8 Nigerian naira which is equivalent to 33.1 US dollars and we estimated that persons on a minimum wage would spend 29% of their monthly income on the procurement of insulin.

**Conclusions:**

Health related costs, age, duration of DM and insulin associated side effects are some of the factors implicated in adherence to insulin prescription.

## Background

The prevalence of diabetes mellitus (DM), is on the rise worldwide and this is more so in the developing countries which unfortunately are already overburdened by a high disease burden arising from communicable diseases. It is estimated that by the year 2030, over 70% of people with diabetes will reside in developing countries [1]. The burden of DM is unacceptably high in the developing countries of the world with oft documented high morbidity and mortality rates. Good glycaemic control is of utmost importance in reducing the burden of disease attributable to diabetes mellitus. Optimal management of DM is a key factor in reducing the afore mentioned scenario. Management of DM includes the employment of non pharmacological and pharmacological interventions of which insulin therapy plays a prominent role. Insulin therapy is used in the management of diabetes mellitus of all types and the need for insulin depends on the balance between insulin secretion and insulin resistance. Effective usage of insulin in the management of glycaemia remains a challenge in developing countries like Nigeria. In a Nigerian report, the percentage of persons with type 2 DM documented to be on insulin therapy was 15.4% with 5.4% on insulin solely and 10% on varying combinations of insulin and oral hypoglycaemic agents [2].

In most developing countries, the mainstay of insulin delivery is single or multiple daily subcutaneous injections and commonly used insulin delivery devices include insulin syringes and pens. Insulin therapy remains widely unacceptable amongst patients with DM and reasons for this scenario range from needle phobia, costs and inconvenience of daily injections.

There is a plethora of terminologies used with reference to medication -taking behaviour and these include “adherence”, “concordance”, “persistence” and “compliance”. Adherence is defined as ‘the extent to which a person’s behavior (in terms of taking medications, following diets, or executing lifestyle changes) coincides with medical or health advice [3]. Rates of refilling for prescriptions have been used as a method of measuring adherence and is also another means of testing “persistence”. Persistence is a relatively recent terminology that describes the duration of continuous medication use and is of limited value in clinical practice. This is because it refers more to how frequently a patient will collect a prescription for a certain treatment rather than whether it is actually taken or not [4]. Compliance is defined as: “the extent to which the patient follows the health professionals’ advice and takes the treatment” [4]. As opposed to adherence, compliance is rather one of passivity on the part of the patient and does not take cognisance of the patient having a say in the management of the disease condition. Concordance is defined as: “agreement between the patient and healthcare professional, reached after negotiation that respects the beliefs and wishes of the patient in determining whether, when and how their medicine is taken and describes an agreement drawing upon the experiences of both provider and patient [4]. We however decided to use the term adherence in our Report especially as it is more suitable to our Study compared to the other stated terms.

The objective of this Report is to document the pattern of insulin prescription, regimen, costs and attendant problems associated with its use. We also attempt to evaluate for factors affecting insulin adherence.

## Methods

This was a Cross sectional observational study carried out at the Diabetic Centre of the Lagos State University Teaching Hospital, (LASUTH), Lagos State. Persons living with diabetes mellitus who were on insulin treatment (for at least a period of one month) solely or in combination with oral hypoglycaemic agents and who gave their consent were recruited.

Ethical approval for the study was obtained from the Lagos State University Teaching Hospital.

Participants provided information on their diabetes history, management and problems associated with management. The type of insulin in use, the number of daily injections, issues with adherence were ascertained from the Case folders, patient interview and prescriptions. Gainful employment where present was noted and the monthly income for those who earned a salary was documented.

Clinical examination was carried out basically to determine the anthropometric indices.

Long term and short term glycaemic control was evaluated for using glycosylated haemoglobin and fasting plasma glucose assessment respectively.

Good long term glycaemic control referred to HbA1c level of ≤6.5% [5].

Short term glycaemic control referred to FPG levels of ≤110 mg% [5].

Type 2 DM-Patients were classified as having type 2 diabetes mellitus using clinical criteria such as a present/prior history of usage of oral hypoglycaemic agents or usage of combination of insulin and the oral hypoglycaemic agents [6].

Type 1DM- This referred to patients who are presently on insulin and have been insulin requiring since diagnosis [6].

Information on insulin adherence was provided by the patients and their family members.

Insulin adherence was defined as “taking medication as prescribed and/or agreed between the patients and the health care provider over a period of one month”.

The “adherence rate” for the Study subjects referred to the proportion of patients who used insulin as prescribed over a period of one month.

Non adherence referred “not taking medication as prescribed and/or agreed between the patients and the health care provider over a period of one month”.

Insulin costs (direct cost) per month was determined through patient’s interview. Information on the number of vials or penfills of insulin and costs per month was obtained from the patient. Costs of insulin were calculated in Nigerian Naira (NGN) and converted to US dollars (USD) and the prevailing currency exchange rate was 150NGN to 1USD.

The proportion of income spent on insulin was calculated by the following formula:

(1) $$\frac{\text{Mean insulin costs of those earning an income}*100}{\text{Prevailing Nigerian Minimum wage}}$$

(The prevailing minimum wage was 18,000 NGN or 120 USD)

SPSS was used for statistical analyses and a p value of ≤ 0.05 was deemed to be significant.

## Results

Of the 160 Respondents in this Report, females were 104 (65%) and males were 56 (35%). The mean age in years was comparable in both genders (Females −53.6 (13.5) Vs Males −52.1 (13.8), p = 0.5).

Persons with type 1DM were 31 and type 2DM were 129 in number accounting for 19% and 81% respectively of the Study population. About half (53%) of the persons studied were gainfully employed and earned an income. Well over half 122 (76.3%) of the subjects were married, 23 (14.4%) were divorced or widowed and 15 (9.3%) were single. A breakdown of the cadre of educational status is as listed: Non-literate 14 (8.7%), Primary education-44 (27.5%), Secondary education-51 (31.9%), Tertiary education −51 (31.9%).

The Clinical features of the Study population are shown in Table 1.

**Table 1:** Clinical characteristics of the Study Subjects

**Parameter**	**Mean (SD)**	**Range**
Age (years)	53 (13.6)	18.29
BMI (kgm2)	27 (5.7)	15.5-53.3
Waist circumference (cm)	77.8 (66.8)	38-133
Duration of DM (years)	10.3 (9.8)	0.4-35
Duration of insulin use (years)	4.2 (5.9)	0.1-31

### Treatment types, administrative devices and dosing frequency

Prior to insulin treatment, 126 (98%) of the Study subjects were on oral hypoglycaemic agents and 3 (2%) resorted to use of herbal remedies. With insulin treatment, the pattern of treatment was such that of the patients with type 2 DM, 118 (91%) were on combinations of insulin and oral hypoglycaemic agents and 11 (9%) were on sole insulin treatment.

**Figure 1:**
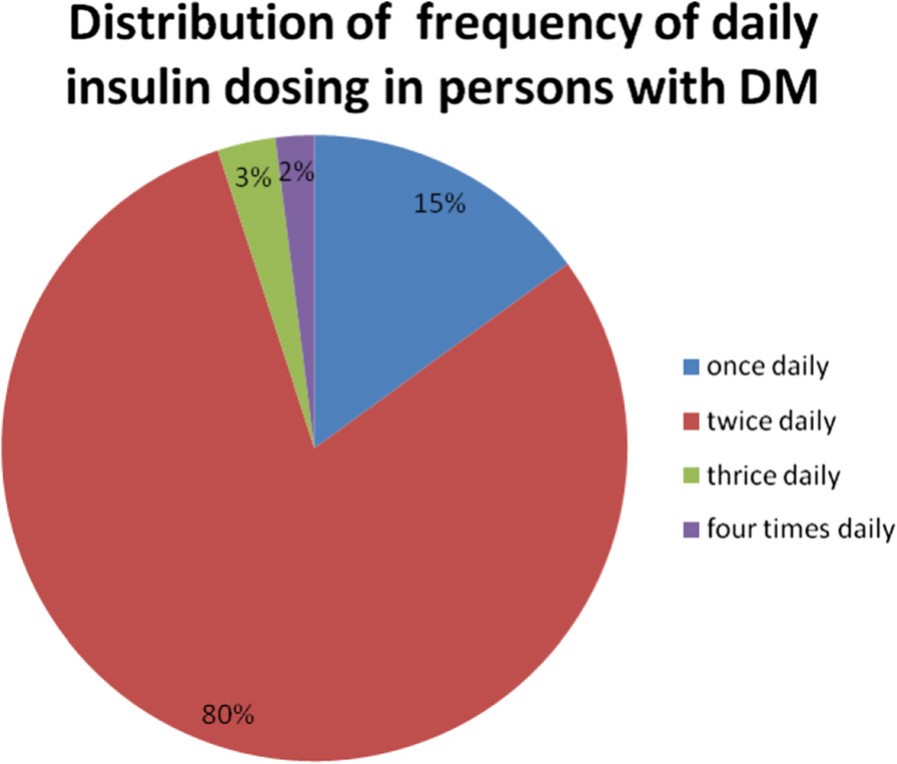
Distribution of frequency of insulin dosing in persons with DM.

Insulin dosing regimen ranged from once to four times daily. The distribution of the dosing frequency of insulin is shown in Figure 1.

Insulin administrative devices used were Insulin syringes-113 (71%) and Insulin pens 47 (29%). The large majority-150 (94%) of the subjects self inject insulin. A total of 90-(56%) of the subjects practised self home glucose monitoring.

Human Insulin was the commonly used insulin with premixed preparations taking the lead. The pattern of insulin use is shown in Table 2.

**Table 2:** Pattern of insulin use

**Type of insulin**	**N (%)**
**Human Insulin**	
Fixed dose combination (30/70)	130 (81%)
Lente Insulin	14 (8%)
NPH Insulin	9 (6%)
Regular Insulin	3 (2%)
**Insulin Analogues**	
Glargine	4 (3%)

**Figure 2:**
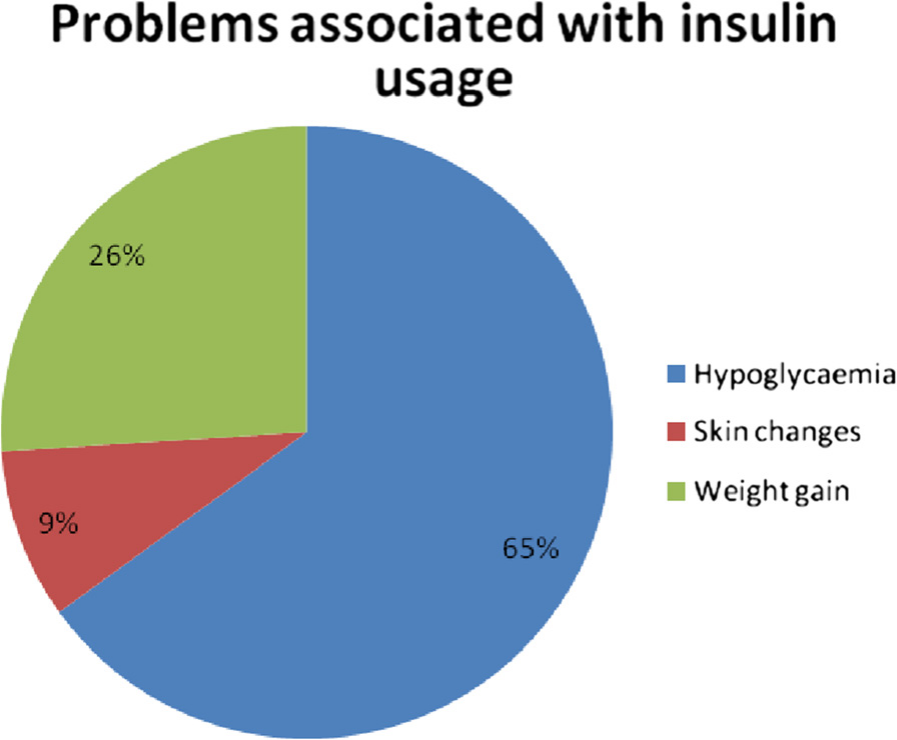
Problems associated with insulin use.

Hypoglycaemia was the most frequently documented problems encountered by persons on insulin. Figure 2 depicts the frequency of problems associated with insulin usage.

### Insulin adherence

Insulin adherence was noted in 123-77% -of the Study subjects and this was comparable between persons with type 1 DM and those with type 2 DM. Long term good glycaemic control was 52 (32%) of the patients. The mean glycosylated haemoglobin values were significantly higher in those who admitted to non insulin adherence compared to those who adhered to their insulin regimen (9.7% (2.3) Vs 8.6% (2.1), p = 0.01).

For the respondents who could not adhere to their insulin regimens, reasons adduced for this scenario include the following; High costs-15(41%), inconvenience −15(41%) and needle pain-79)18%. 77 (48.1%) persons experienced problems with insulin injections and these were Hypoglycaemia-51-(66.2%), Weight gain-19-(24.6%) and Skin changes, hyperpigmentation and skin atrophy −7-(9.2%)

A greater proportion of persons who self injected insulin adhered to insulin prescriptions compared to those who did not adhere. The mean indices of short and long term glycaemic control were noted to be higher in persons who did not adhere to insulin prescriptions. These results are shown in Table 3.

**Table 3:** Comparison of clinical and biochemical parameters in persons who adhered and those that did not adhere to prescribed insulin regimens

**Variable**	**Insulin adherence**	**Insulin non adherence**	**p**
Mean Hba1c (%)	8.6 (2.5)	10.1 (2.5)	0.008
Fasting plasma glucose (mg%)	165.5 (83.5)	205.6 (89.8)	0.02
Previous DM hospitalization	83 (68%)	18 (48.6%)	0.03
Gainfully employed	63 (52%)	22 (60%)	0.4
Family hx of DM	67 (55%)	22 (60%)	0.5
Self insulin injection	118 (96%)	32 (87%)	0.03
Self glucose monitoring	72 (59%)	18 (49%)	0.2

Shorter duration of DM and older age were possible predictors of insulin adherence. These results are shown in Table 4. Insulin Adherence was comparable between persons who were educated and those who had no form of literacy (85% vs 76%, p = 0.3).

**Table 4:** Evaluation of possible predictors of insulin adherence

**Variable**	**Exp (B) interval**	**95% Confidence interval**	**p**
Duration of DM	0.9	0.88-0.99	0.03
Age	1.1	1.06-1.73	0.01
Educational status	1.2	0.23-6.23	0.8
Type of DM	2.7	0.8-9.25	0.1
Self payment	0.7	0.29-1.86	0.5
Gender	1.44	0.5-3.53	0.4
Gainful employment	1.1	0.5-2.4	0.7

### Insulin costs

The mean costs of insulin estimated for the Study subjects was Nigerian Naira (NGN) 4534.9 (4742.7) and USD29 (30). The calculated median insulin cost was NGN 3, 400(USD21.6) and range were NGN 1120 to NGN 40, 000 and USD 7.1 to USD 254.6 respectively. About half-94(58%) of the Study subjects paid for their insulin themselves, -13(8%) had their insulin paid for by their parents (this group of patients had type 1 DM), -10(6%) had their insulin paid for by relations and 43-(27%) had their insulin paid for by their children.

For those who earned a salary, the proportion of income spent on insulin was determined to be 29%. A summary of the income and costs of insulin of those who paid for their insulin themselves are shown in Table 5.

**Table 5:** Income and Insulin costs for self paying patients

**Variable**	**Mean (SD)**	**Median**	**Range**
Income (NGN)	63,319 (1386.1)	30,000	5000-1500000
Income (USD)	403 (8.7)	190.9	31.8-9548
Insulin costs (NGN)	5212.8 (5878.8)	3650	1120-30,000
Insulin costs (USD)	33.1 (37.4)	23.2	7.1-190.9

### Comparison of some clinical parameters with respect to insulin use between persons with type 1 and those with type 2 DM

Some clinical parameters compared between persons with type 1 and those with type 2 DM are shown in Table 6.

**Table 6:** Comparison of clinical parameters between persons with type 1 and those with type 2 DM

**Variable**	**Type 1**	**Type 2 DM**	**p**
Insulin adherence	22 (71%)	101 (78.3%)	0.3
Self injection of insulin	30 (97%)	120 (93%)	0.7
Self blood glucose monitoring	19 (61%)	71 (55%)	0.5
Mean cost of insulin per month (NGN)	3664.5 (1606.3)	4745.7 (5210.1)	0.04
Mean cost of insulin per month (USD)	23.3 (10)	30.2 (33.2)	0.04
∗Hospitalization frequency for poor glucose control	25 (80.6%)	76 (58.9%)	0.02

∗Proportion of persons with previous hospitalizations for poor glucose control.

**Figure 3:**
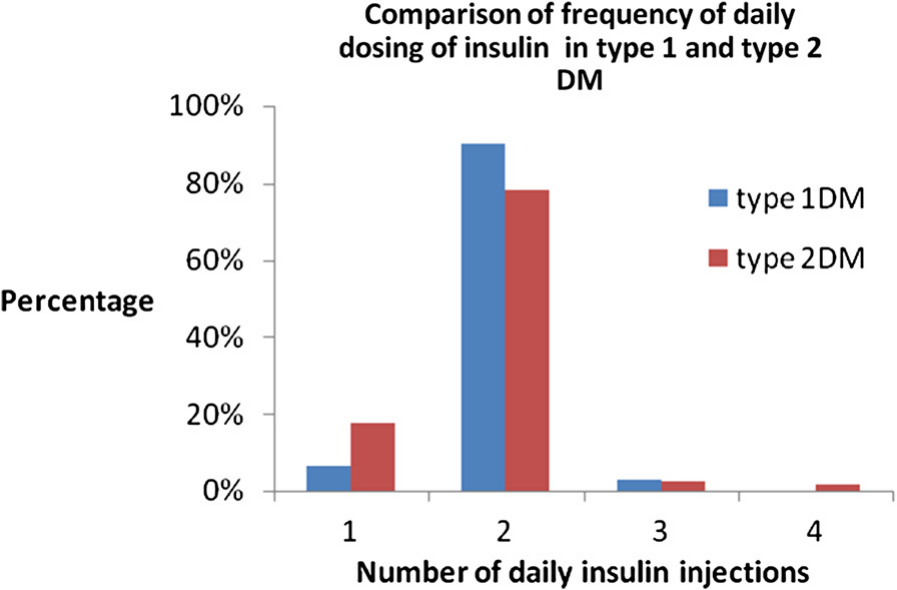
Comparison of frequency of daily dosing of insulin in type 1 and type 2 DM.

In the comparison of frequency of insulin injection in persons with type 2 DM and those with type 1 DM, we noted that more people with type1 DM injected insulin twice a day compared with persons with type 2 DM. In our Report we observed that only persons with type 2 DM were on a four times a day insulin regimen. These results are shown in Figure 3.

## Discussion

The role of insulin in the management of diabetes mellitus cannot be overemphasized and people with diabetes use combinations of different types of insulin to better control and manage their condition [7,8]. In this Report, we note that the majority of persons with type 2 DM who are on insulin therapy use insulin in varying combinations with oral glucose lowering agents.

A third of the respondents reported omitting insulin injections with the commonly documented reason for this being the associated high costs of insulin. Often described barriers to use of insulin include fear of injections and hypoglycemic events, burden of injections, inconveniences associated with its use [9,10]. The issue of costs is hardly reported although in sub-Saharan Africa poor accessibility to healthcare is a problem. In a Nigerian study on adherence to medications in persons with DM, the issue of high costs of medication was brought to the forefront [11].This observation was supported by the findings in the Diabcare Africa Study which was carried out in six countries in sub-Saharan Africa[12]. In Nigeria, the minimum monthly wage is 18,000 naira (120USD), and the mean costs of insulin for those who paid for their insulin themselves -who incidentally had type 2 DM- was 5212 (33.1USD). We deduce that a person on minimum monthly wage would spend about 29% their monthly income on the procurement of insulin. In this Report, all Respondents practiced “out of pocket” payment for healthcosts. (The large majority of persons attending the government hospitals in Nigeria are not on health insurance schemes). Insulin costs incurred by persons with type 2 DM were however higher than those incurred by persons with type 1 DM. Reasons for this scenario may be partly explained by the observation that some respondents with type 2 DM injected insulin four times daily and also used insulin analogues which are more expensive than human insulin. Insulin analogues are not readily accessible in our practice and are prescribed only by Endocrinologists.

The combination of prandial and basal insulin clearly results in better glycaemic control and less glucose variability [13].Multiple insulin dosing administration commencement in our patients with DM depends on the degree of hyperglycemia and the patient’s acceptance of multiple daily injections. In our practice we observe that persons with type 2 DM are often more motivated than those with type 1 DM in the acceptance of usage of multiple daily insulin injections. Cost and lifestyle limitations are multiple insulin injection related issues of which patients with type 2 DM are likely to be more empowered than persons with type 1 DM to take in their stride. Our findings of multiple daily insulin injections in persons with type 2 DM are most likely due to the aforementioned reasons. The health Insurance scheme is at best sub-optimal in our practice and none of the patients seen in our clinic on insulin is on a health insurance scheme.

Insulin adherence is not widely studied in sub-Saharan Africa and thus factors associated with adherence are often not objectively substantiated for. We report an insulin adherence rate of 77% and the rates were comparable- between persons with type 1 and those with type 2 DM. In a review on insulin adherence in type 2 DM, adherence rates were found to be lower for insulin use than for OHA use and ranged between 73–86% [14]. The factors that significantly affected insulin adherence in our Report included previous hospitalization self insulin injecting practices, duration of DM and age. The Respondents who admitted to previous DM related hospitalizations obviously did not want a repeat episode hence tended to adhere to prescribed medications for DM. Self insulin injection was practised by a great majority of respondents and this affected insulin adherence positively. We note also that the lesser the duration of DM, and the older the age of the respondent, the more likely the chances of adherence to insulin therapy. Literacy was not found to have an impact on adherence and we document comparable adherence rates between persons who are literate and those with no form of literacy.

Insulin syringes remain the commonly used delivery devices and the reason for this is ready accessibility. The insulin pens which are more convenient to use are at present not readily available to most persons with DM as only a third of the Respondents used them. Continuous subcutaneous insulin infusion as a means of insulin administration is virtually absent in our practice and may even prove to be unaffordable even if available.

Hypoglycaemia and weight gain are issues associated with insulin use and may be obstacles to the use of insulin [15]. In our Study, hypoglycaemia was noted in well over half of the study subjects whilst weight gain and skin changes were reported by a third of the respondents. In a Report on insulin omission in women with type 1 DM, weight gain was noted to be a reason why 31% of the Study participants reported intentionally insulin dose omission [16].

The use of insulin is important to effectively control the disease process in patients with diabetes mellitus and insulin adherence has been especially proven to be associated with good long term metabolic control [17]. We have shown that long term glycaemic control in persons with DM tends to be poorer in persons who show non adherence to prescribed insulin.

## Conclusion

From the foregoing, we note that the factors affecting insulin adherence include modifiable and non modifiable factors thus adherence interventions might help improve this all important aspect of healthcare.

### Limitation

Categorical endpoints were not used to determine insulin adherence.

## Competing interests

The authors declare that they have no competing interests.

## Authors’ contributions

AOO was responsible for the Study design, Funding, Data collation, Statistical analyses and Drafting of the manuscript. SFK was responsible for the Study design, Funding and Drafting of the manuscript. All authors read and approved the final manuscript.
